# Unlocking All-Solid Ion Selective Electrodes: Prospects in Crop Detection

**DOI:** 10.3390/s22155541

**Published:** 2022-07-25

**Authors:** Jiawei Zhai, Bin Luo, Aixue Li, Hongtu Dong, Xiaotong Jin, Xiaodong Wang

**Affiliations:** 1 Research Center of Intelligent Equipment, Beijing Academy of Agriculture and Forestry Sciences, Beijing 100097, China; zjw2933795846@163.com (J.Z.); luob@nercita.org.cn (B.L.); liax@nercita.org.cn (A.L.); donght@nercita.org.cn (H.D.); jinxt@nercita.org.cn (X.J.); 2 College of Mechanical and Electrical Engineering, Fujian Agriculture and Forestry University, Fuzhou 350108, China

**Keywords:** all-solid-state ion-selective electrode, crop ion detection, ion selective electrode potential method, sensor miniaturization

## Abstract

This paper reviews the development of all-solid-state ion-selective electrodes (ASSISEs) for agricultural crop detection. Both nutrient ions and heavy metal ions inside and outside the plant have a significant influence on crop growth. This review begins with the detection principle of ASSISEs. The second section introduces the key characteristics of ASSISE and demonstrates its feasibility in crop detection based on previous research. The third section considers the development of ASSISEs in the detection of corps internally and externally (e.g., crop nutrition, heavy metal pollution, soil salinization, N enrichment, and sensor miniaturization, etc.) and discusses the interference of the test environment. The suggestions and conclusions discussed in this paper may provide the foundation for additional research into ion detection for crops.

## 1. Introduction

Modern technology has been able to accurately detect inorganic ions in plants [1,2]. Inorganic ion detection has been used to detect plant nutrient ion concentrations, heavy metal ions in heavy metal stress, and plant pollution control results.

Many inorganic ions are essential constants in many aspects of plants, such as photosynthesis, osmotic regulation, phloem transport, and cell electrochemistry [3,4]. K^+^ provides osmotic regulation and participates in activating enzymes and plant metabolic processes [5]. Ca^2+^ and Mg^2+^ take on many functions in plants, such as the stability of cell membrane structure, signal transmission, participation in secondary metabolism, etc. [6,7,8]. N is absorbed by crops in the form of ammonium nitrogen and nitrate nitrogen and plays an important role in the growth of crops. In addition, the study in [9] showed that adding N and P nutrients could effectively alleviate heavy metal damage, to a certain extent. On the other hand, plants are highly sensitive to toxic metal ions. A small amount of toxic metal ion stress can induce varied physiological symptoms in plants. Excessive heavy metal ions have inhibited seed germination and seedling growth, destroyed antioxidant enzymes and membrane systems, induced chromosomal aberration, and even led to plant death in serious cases [10,11,12]. Some heavy metals, as trace elements, are necessary for plant growth and development and play an important role in plant metabolism [13]. Ion concentration detection under environmental stress such as drought and salt has also been an agricultural research direction [14,15,16,17,18,19,20,21]. The aforementioned evidence suggests the importance of ion detection.

There are many methods for ion detection, including spectrometry, mass spectrometry, chromatography, spectrophotometry, and electrochemical analysis [1,22,23]. Current ion-selective electrodes (ISEs) showed good performance in ion selectivity, with a low detection limit and a rapid response [24]. Meanwhile, ISE has been widely used due to its advantages of simple fabrication, low cost, and strong portability. In addition, the multi-array ISEs are easy to implement, which facilitates the simultaneous detection of ion concentrations across multiple channels. The ASSISE overcomes the disadvantages of glass microelectrodes, such as inflexibility and fragility, and could become the mainstream in ion detection in the future. Rapid ion detection has been widely used in biomedical, agricultural, environmental, and industrial analyses [25]. This review introduces the principle and current research progress of ASSISE and the prospect of future development in crop detection.

## 2. Advances in All-Solid-State ION-Selective Electrodes

### 2.1. Principle of Ion Selective Electrode Potential Method

The ISE method has been used in vegetable detection since 1992 [26]. ISE potential method is a branch of potentiometric analysis, which is based on the functional relationship between electrode potential/electromotive force and ion activity/concentration to quantitatively analyze and determine the content of ion in solutions [27] and is based on the Nernst response principle. ISE is a kind of electrode that is used to measure the ion concentration in a solution based on the electrochemical reaction mechanism. A chemical response signal is converted to electrical signals according to the potential analysis method and experiment technology in the electrochemical analysis.

**Figure 1:**
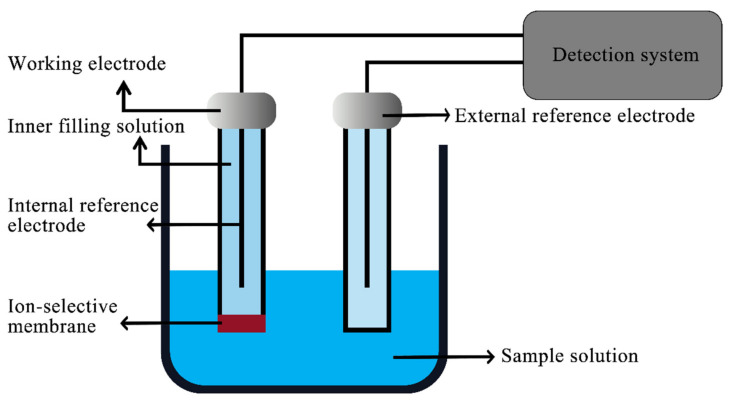
Schematic diagram of ion electrode.

The structure of an ionic electrode is shown in Figure 1. The ion electrode can be divided into a solid electrode and liquid electrode, depending on whether it contains liquid. As compared to the traditional liquid electrode, the new solid-state electrode does not contain liquid, so the detection process will not result in evaporation or pollute the reagent. This type of detection is also easy to miniaturize, and the electrode can be reused.

Electrochemical sensors also have other detection principles and methods. These sensors are roughly divided according to their working mechanism, such as: potentiometric (potential), voltammetry/amperometry (current), and electrochemical impedance spectroscopy (EIS) methods. The potentiometric method is based primarily on the Nernst response. The ion species of ASSISEs depends on the species of ionophore, which is based on the potentiometric method [28,29,30]. The voltammetry/amperometry method applied voltage to obtain current feedback. This is the result of electrolysis by oxidation or reduction at the working electrode [31], such as cyclic voltammetry (CV) and differential pulse voltammetry (DPV) [32,33]. Linear scan voltammetry measures the performance of electrodes [34]. EIS measures the response of a circuit or electrochemical system to a low applied AC potential [35]. The EIS method relies on electron transfer between the electrolyte and the electrode surface [36,37]. Considering the practical application of agriculture, the detection method of the potentiometric sensor is more suitable for long-term continuous monitoring, while other voltammetry/amperometry and EIS detection methods are only suitable for instantaneous ion concentration detection.

### 2.2. Principle of All-Solid-State Ion-Selective Electrode

**Figure 2:**
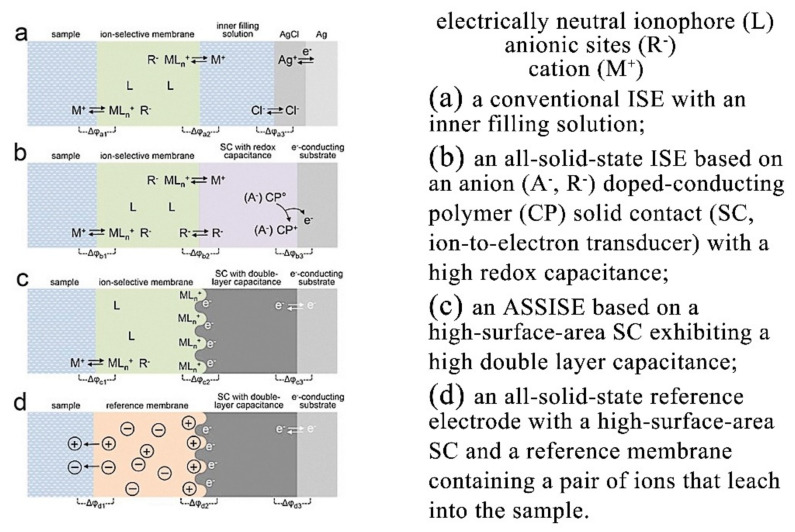
The schematic diagram of the ASSISE transduction mechanism. “Reprinted/adapted with permission from Ref. [41]. 2016, Elsevier”. More details on “Copyright and Licensing” are available via the following link: https://s100.copyright.com/order/698751e4-b909-49d0-8e4f-97d24ddb1557 (accessed on 6 June 2022).

The ASSISE replaces the liquid-filling-liquid and the internal reference electrode in a liquid ISE with a solid conductive matrix. The schematic diagram of the ASSISE transduction mechanism is shown in Figure 2. ASSISEs are roughly categorized as wire-coated electrodes, solid contact (SC) ISEs, monolithic ASSISEs, and non-polymer membrane ISEs [38,39,40].

The SC has the function of ion-electron conduction. In order to improve the potential stability and make the ion detection limit lower and the sensitivity higher, many electrochemical materials with ion-electron conduction properties are used as the SC layer. Examples include Ag/AgCl, hydrogels, redox polymers, self-assembled monolayers, and porous carbon materials [42,43,44]. High capacitance is essential for good performance in ASSISEs. Micro/nanostructures have been an effective strategy to improve capacitance [45]. The study in [46] presents a comparative examination of all ASSISEs based on different multi-walled carbon nanotube ionic liquid nanocomposites. It was shown that the type of multi-walled carbon nanotube (MWCNT) used for nanocomposite preparation intrinsically affected electrode performance, and the structure (diameter, uniformity) of the carbon nanotubes (CNTs) used for nanocomposite preparation was important. The study in [29] investigated the design of all-solid-state Cl^−^ and NO_3_^−^ ISEs using electrodeposited polyallylamine-MnO_2_ nanocomposite layers as internal intercalation materials. The effect of the SC on the sensor capacitance was measured more accurately by EIS [47,48]. Chronopotentiometry can be used to evaluate the potential stability of SC [49]. There were also tests conducted related to the effects of O_2_, CO_2_, light, and water layers to verify the stability of the SC [50]. The study in [41] discussed methods for solid-state ion-exchange electrodes and reference electrodes to improve reproducibility, EMF response stability, lower detection limits, and novel sensor designs.

**Figure 3:**
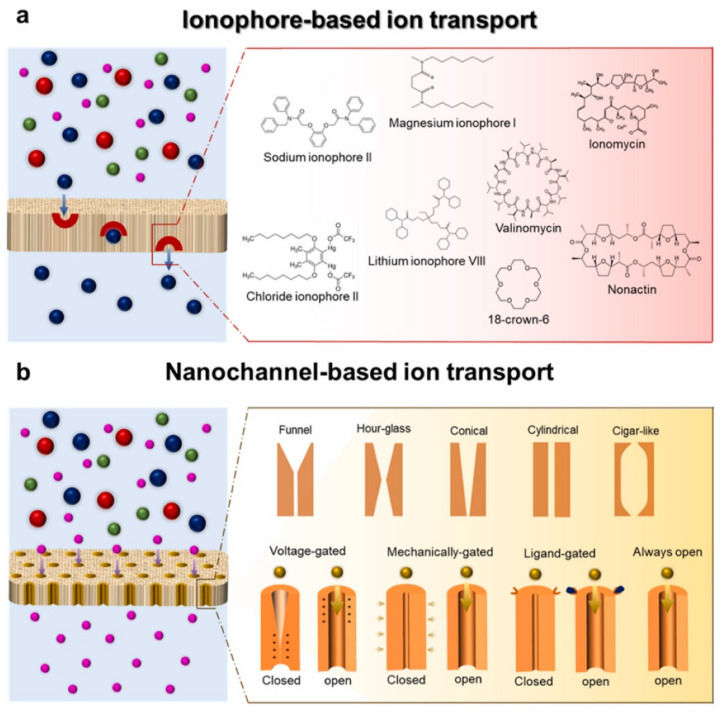
Schematic diagram of two strategies for ion-selective membranes. (**a**) Ionophore-based ion transport; (**b**) nanopores-based ion transport. “Reprinted/adapted with permission from Ref. [51]. 2021, Elsevier”. More details on “Copyright and Licensing” are available via the following link: https://s100.copyright.com/order/cdd17eeb-cdaa-4853-8367-e7224524dafd (accessed on 6 June 2022).

For ion-specific recognition, the potentiometric measurements of ion activity based on ion acceptors/ionophores have existed since 1960 [51]. Wilhelm Simon et al. had made outstanding contributions to the research on ionophore [52,53]. Two general strategies have been applied to design and fabricate ion-selective membranes using specific ionophores/ion acceptors or nanochannels/nanopores in different matrices (Figure 3) [51,54]. The study in [54] summarized the methods and materials for the preparation of ion-selective membranes.

## 3. Key Performance Indicators of All-Solid-State Ion-Selective Electrode

ASSISE parameters that describe performance include stability, slope, detection limit, working life, and so on. Special emphasis has been placed on the response range and service life in crop testing. Firstly, due to the variety of crops, the concentration range of various ions is very wide. Secondly, the service life is of great significance for the electrode price and promotion. At the same time, it should be considered as to whether the electrode production process is complex, which could also affect the large-scale promotion of ASSISE.

### 3.1. Stability

**Figure 4:**
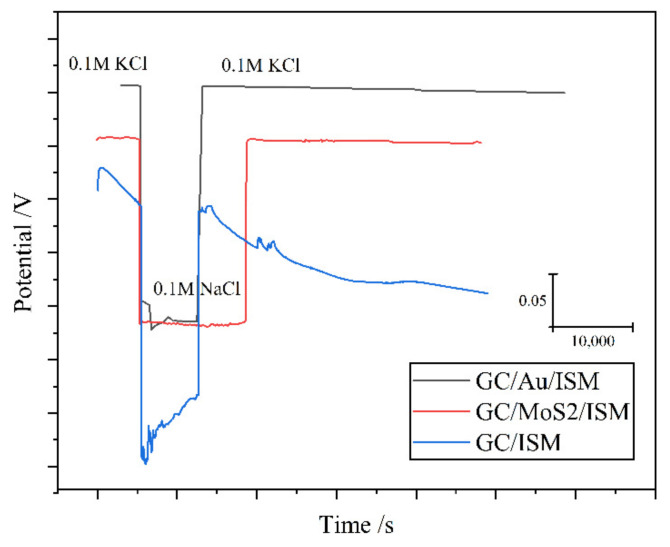
Diagram of water-layer test [56,57] (GC/ISM stands for glass carbon electrode without SC, and is the author’s experiment).

Stability consists of reliability and reproducibility. The electrode reliability indicates that the electrode response potential can be measured steadily and continuously without the loss of fidelity over a period of time. Reproducibility refers to the degree of reproducibility of the electrode potential after repeated alternating measurements in a variety of solutions with different concentrations. Water-layer testing was determined to be highly representative. A thin layer of water was formed between the polymer film and the inner electrode, which can result in the potential instability of an electrode [46]. The performance of the hydrophobicity of SC in the water layer test deserves attention. In addition, the paper [55] proves that the hydrophobicity of graphene is negatively related to its capacitance, so it is necessary to find a balance between hydrophobicity and capacitance for the SC. Figure 4 shows the K^+^ ASSISEs water-layer test diagram of Au and metal sulfide as SC.

### 3.2. Slope and Detection Limit

This section uses Cl^−^, K^+^, Ca^2+^, and so on as examples to understand the research status of the ASSISEs’ response slope and detection limit.

For negative ions, the response slope was opposite to that of positive ions. Although Cl^−^ has had little effect on plants in culture media [58], there have been many research methods (industrial, etc.) that have been used as references. The study in [46], based on multiwalled carbon nanotubes–ionic liquid trihexyltetradecylphosphonium chloride nanocomposite, was prepared by nitrate ISE. It had a response slope of −57.1 mv/decade and a detection line of 10^−6.3^ M. A Cl^−^ ISE was fabricated based on nanowires made of poly(3,4-ethylenedioxythiophene). It had a response slope of −58.1 ± 1.5 mv/decade and a detection line of 10^−5^ M [59]. However, detection methods for Cl^−^ have been focused on cyclic voltammetry for detection [60,61,62,63]. There have also been studies based on amperometric detection (potentiostatic method) to detect Cl^−^ concentrations [64,65,66]. In fact, this classification method has been conducted in pH detection, pollutant detection, biomolecular detection, and so on [67,68,69].

Table 1 introduces the current development of K^+^ ASSISE. Table 2 describes the current research on Ca^2+^. The studies in [70,71,72,73] proposed that a pulsed-time potentiometric measurement of Ca^2+^ could reveal an interesting super-Nernstian response characteristic. The study in [74] introduced the super-Nernstian response in a variety of situations, including the aforementioned electric current pulses on the membrane, resulting in an ion flux of defined magnitude and duration. The detection range of K^+^ and Ca^2+^ has been used to verify whether they met the requirements of crop detection.

Hydroponics are often used in agriculture. The general culture medium concentration in a hydroponic system is shown in Table 3. According to Table 1, Table 2 and Table 3, the main ion concentration was approximately 10^−3^ M, while the ASSISE detection range based on the Nernst response was generally 10^−5^–10^−1^ M.

However, according to the ion concentration in crops, the K^+^ concentration in soybean leaves reached 17.74 mg kg^−1^ [90] (based on 90% water content of plants, the approximate concentration was 4.5 × 10^−5^ M). According to the study in [91], the concentration of ions in a crop was used to determine whether a pathogen had invaded. According to the analysis of tomato leaf midribs, leaf lamina, and roots, the concentration range of K^+^ was approximately 20,000–80,000 mg kg^−1^ (0.05–0.2 M), and the concentration range of Ca^2+^ was approximately 10,000–25,000 mg kg^−1^ (0.025–0.06 M) [91]. The shoot K^+^ concentrations of six selected barley varieties examined in [18] ranged from 200 to 1600 μmol/g (0.02–0.16 M) under different stressors. Therefore, ASSISEs met the requirements for crop ion detection.

Another element necessary for crop growth, phosphorus (P), has been considered [92,93,94]. There have only been a few studies on phosphorus sensors. For example, in [95], the electrochemically modified phosphate ion sensor based on a nickel metal electrode had a slope of −81.0 mV/decade (due to the different types of nickel oxide and phosphate compounds, it had a mixed potential response), the detection limit was 10^−5^–10^−1^, and the electrode had good stability that could be measured continuously for 24 h. The service life was more than four weeks. There were also electrodes chemically modified with a cobalt oxide film to determine phosphorus in diesel fuel by the potentiometric method [96].

Trace elements such as Cu, Fe, and others have been examined for detection range. In a fitted nonlinear curve of a Cu^2+^ selective electrode, the detection range was approximately 10^−7^–10^−4^, and this electrode had a service life of more than one year [97]. The linear response range of Cu^2+^ ASSISE based on xylyl benzene (N,N-diisobutyl dithiocarbamate) was 10^−6^–10^−1^ M [98]. According to the study in [99], a screen-printed Fe^3+^ ISE had a detection range of 10^−7^–2.5 × 10^−2^ M. However, the detection range of the liquid to be tested could not be satisfied. The study in [100] indicated that the pulsed electrode technique reduced the detection limit to 10^−7^ M [101], whereas the tuned galvanostatic polarization method reduced the detection limit to 10^−10^ M [102,103]. These examples provided directions for reducing the detection limit of ASSISE.

### 3.3. Electrode Life

The service life of ASSISEs is directly linked to the cost of use. The paper-based cost is low, but most are disposable products. According to the study in [80], it was impossible to even test for hours. A Ca^2+^-selective electrode based on an inorganic redox buffer of Ag@AgCl/1-tetradecyl-3-methylimidazolium chloride had a good potential life of 30 days [83]. The Ca^2+^ gold electrode of n-phenyl-ethylenediamine-methacrylamide had a lifetime of 3 months [87]. There was also a Ca^2+^ ASSISE with a substantially constant-response slope-and-detection limit for 60 days of continuous use [88]. However, most studies have rarely mentioned the service life of ASSISE.

## 4. Prospection of Crop Detection Applications

### 4.1. Prospects of Ion Concentration Detection In Vitro

#### 4.1.1. Prospects of Crop Hydroponic Applications

**Figure 5:**
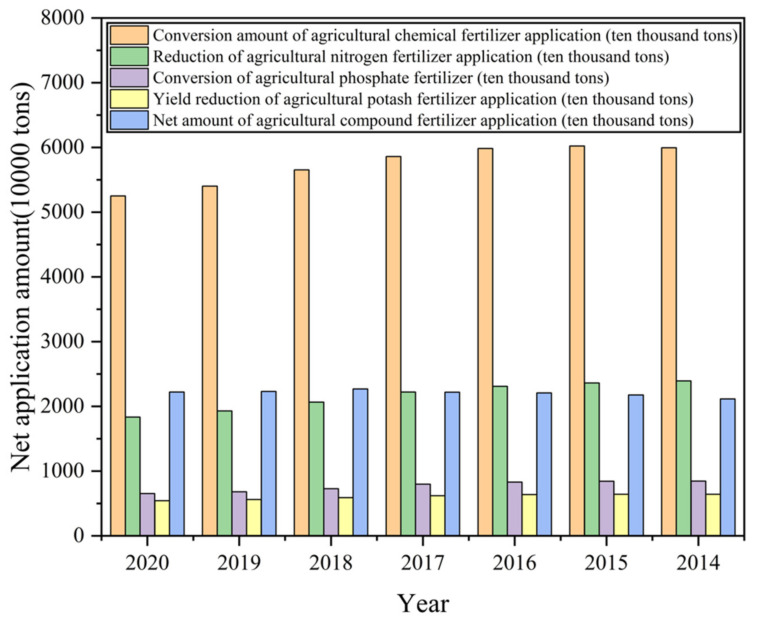
Chart of fertilizer use in China [104].

Food is a necessity for survival. The use of chemical fertilizer has always been extensive in the agricultural industry. Figure 5 shows the use of chemical fertilizer in China. In the process of planting crops, the detection of the nutrient solution and the ion absorption has a protective cover for normal plant growth and harvest.

Soilless cultivation aiming at improving nutrient efficiency has been the most important cultivation method in facility agriculture. Hydroponics and substrate culture together constituted soilless culture techniques [105]. To accurately control the concentration of the nutrients in a hydroponic solution, it is necessary to have accurate sensors and detection systems. Similarly, it has been shown to be critical to monitor nutrient ion concentrations in crop yields in a hydroponic environment [106,107]. The study in [108] found that the order of the influence of nutrient ions on plant nutrients (total dry weight) for M. officinalis plants in a hydroponic system was N > K = Ca = P > S = Mg. Field ion monitoring systems in greenhouses and plant factories have also been considered [109,110]. Accurate control of nutrient ions has been necessary in large-scale hydroponic systems, so monitoring sensors were key to research [111,112].

For example, N is an important element for crop growth. The forms of N element in the growing environment of crops are NH_4_^+^ and NO_3_^−^ [113]. Much research has been conducted on these two ASSISEs [29,46,114]. An ASSISE based on polyaniline and aniline/2, 5-dimethoxyaniline polymer as SC, was used for the detection of NH_4_^+^: detection range 10^−4^–10^−1^, response slope 54.20–56.59 mV/decade, potential drift ±0.5 mV every 400 s, and a service life of 3 months [115]. According to the study in [116], an ASSISE method was proposed to detect NO_3_^−^ by coulomb signal detection instead of the potential response, which proved its reliability.

Similarly, trace elements also play an important role in crops. An appropriate amount of trace elements is beneficial to crop growth and human health, while excessive amounts will cause harm [117]. For example, Se is an important trace element needed in the human body [118]. The study in [119] determined levels of Se by square wave anodic stripping voltammetry using a glassy carbon electrode modified with gold nanocages/fluorinated graphene nanocomposite. However, Se is toxic when consumed in excess, so the element has often been considered as a cause of water and soil pollution [120,121]. The study in [122] showed that an indium–tin–oxide electrode modified based on Au/ZnO nanocomposites could be used for the determination of Se(IV) in water by square wave anodic stripping voltammetry. It met the detection limit of trace elements.

#### 4.1.2. Prospect of Crop Ion Detection on Environmental Ion Pollution

In addition, the detection of heavy metals has been broadly researched. Heavy metal pollution not only harms human health, but also reduces crop yields [123]. Heavy metal pollution has been shown to have significant influence on cultivated land and water [124,125,126]. Therefore, the prevention and control of heavy metal pollution is extremely important as it pertains to crop growth, pollution treatment, and the crop itself. Soil salinization and N-enriched environmental pollution should also be considered. The high demand for nitrogen in high-yield grain systems has resulted in large amounts of nitrogen being lost to the environment [127,128]. The study in [129] emphasized that soil salinization inhibited the normal growth of crops and led to the decline in soil fertility and crop yield, so it was important to accurately and rapidly measure the salt content of salinized soil. Therefore, ASSISEs could play an important role in environmental pollution ion detection.

Anthropogenic sources of heavy metals in agricultural soils have resulted from municipal sewage, vehicle exhaust, sewage sludge, pesticides, and fertilizer applications [130,131]. ASSISEs could be used to detect the effect of pollution control. The study in [32] used a gold electrode and a DPV method to test the treatment effect of garlic on environmental pollutants such as mercury, and one relevant algorithm model was established. The study in [132] was based on polyvinyl alcohol/chitosan in a thermally reduced graphene electrode, and lead concentration was measured using square wave anodic voltammetry. The study in [133] developed a printed electrode with hexaammineruthenium(III), and Pb was detected via cadmium square wave anodic stripping voltammetry in natural water samples. ASSISEs have also been used to test the quality of agricultural products [134,135].

### 4.2. Prospects of Ion Concentration Detection In Vivo

To realize crop in vivo detection, it was necessary to miniaturize the sensor. The purpose of electrode sensor miniaturization was to minimize the invasive detection of organisms. Minimally invasive detection is used to ensure edibility of the crops. The plant cuticle has a critical protective effect [136,137]. To limit high intensity ultraviolet radiation; temperature, mechanical, and chemical damage from various plant pathogens; and prevent the evaporation of water and infiltration of pollutants, miniaturized sensors should be considered.

#### 4.2.1. Prospects for Sensor Miniaturization

To reduce the cost, simplify the preparation, and encourage mass production, portable electrode miniaturization is an urgent matter. Thin and thick film techniques have miniaturized the electrode size as well as the whole detection system [138,139]. Furthermore, electrode miniaturization is necessary for the nondestructive testing of plants. At present, ASSISE miniaturization methods have focused on paper and screen-printed electrodes. The interdigitated microelectrode has also been an excellent candidate for battery miniaturization [140].

As paper is readily available, inexpensive, liquid-absorbent, disposable, and biodegradable, paper-based technologies have been extensively studied in the development of potential sensors and their applications in ion determination in a variety of samples [138,141]. Paper-based electrodes have been prepared by printing, coating, cutting, and layering paper [142]. Various types based on printing processes have emerged including wax printing, screen printing, inkjet printing, and aerogel inkjet printing [142,143,144,145]. Other methods have included microwire placement, laser scribing, carbon tape, pencil-drawn, spray-and-spin coating, and sputtering [146,147,148,149,150,151,152,153,154].

Screen printing is a mature technique for making electrodes using materials such as carbon, metal, and nanomaterials on a variety of substrates such as paper, plastic, and ceramics [155,156,157]. The roots of screen printing as a stencil form have been traced back to the ancient Chinese technique of coloring textiles with materials attached to a substrate by a suitable mesh [158]. The study in [30] performed a sweat Na^+^ analysis on a solid ion selective carbon black-modified printed electrode. The study in [159] doped a screen-printed electrode with K^+^ ferrocyanide as a novel solid-state transduction layer in K^+^ ISEs for finite calibration measurements of K^+^. At present, potential sensors based on screen printing electrodes have detected more biological and organic molecules, but fewer inorganic ions [160].

In addition to paper-based and screen-printed electrodes, microneedle electrodes have also been used for the miniaturization of ASSISEs. Microneedle structures have been widely used in medicine due to their technical advantages, such as noninvasive and mild pain [161,162,163]. This has also been accomplished in plant detection [164,165]. The study in [166] used an electrochemical microsensor composed of stainless steel needles to detect Indole-3-acetic acid in soybeans. The study in [167] designed and manufactured a lactic-acid biosensor based on microneedle technology. The same method was commonly used in the detection of biological and organic molecules, but it was less discovery in the detection of inorganic ions.

Research related to 3D printing, graphene paper, and microfluidics has been translated into plant ion detection. The study in [168] showed that a 3D-printed flow manifold could be used for the simultaneous determination of K^+^, Na^+^, Ca^2+^, and Cl^−^ in water, based on flow measurements with ASSISEs, making it an effective tool for modern multicomponent analysis. The study in [169] demonstrated the feasibility of using 3D-printed flow manifolds to determine Na^+^, K^+^, and Ca^2+^ in urine samples. Three-dimensional printing was used to manufacture microneedle electrodes [170]. The work in [171] showed that the flexible laminated graphene paper electrode was an economical and efficient alternative to glassy carbon and platinum-wire electrodes and showed that the performance of the flexible laminated graphene paper, K^+^ selective solid-contact ISE, was comparable to the most advanced solid-contact ISE. Microfluidics, characterized by fluid manipulation in submillimeter channels/reactors, are of great concern in many areas such as polymer synthesis, fine chemical processing, and biomedicine [172,173,174]. The study in [175] introduced a new concept of solid reference electrodes integrated with microfluidic paper-based sampling and was successfully used for ion determination in environmental and clinically relevant samples.

#### 4.2.2. Prospects of In Vivo Detection of Crops

ASSISEs can detect the concentration of ions in crops and plants. Most studies have detected the concentration of ions in the extracted solution after grinding and extracting samples [176,177]. In [86], a rapid, portable, and disposable screen-printed carbon electrode sensor was proposed to detect NO_3_^−^ and Ca^2+^ in sesame leaves, and the method of collecting the liquid to be measured was to crush and press sesame leaves. This paper [178] tested the inhibition of alkali stress on oats and how to improve the ions of various elements by freezing oat samples with liquid nitrogen and then crushing them.

**Figure 6:**
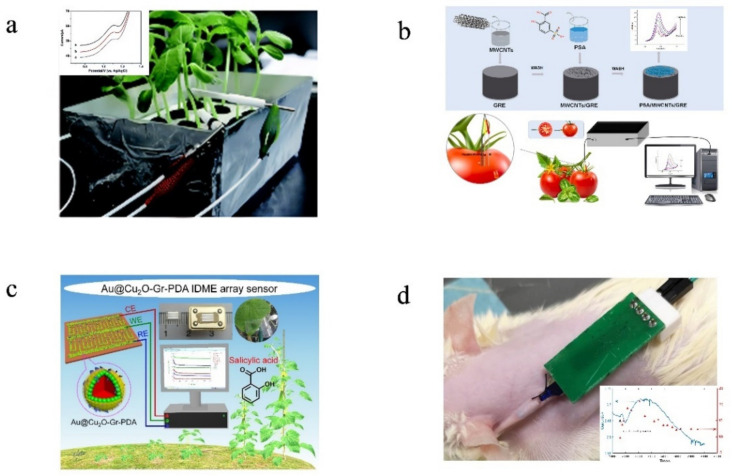
(**a**). Detection of salicylic acid in sunflower seedling stems by needle electrode [189]. (**b**) Detection of L-tryptophan in tomato by needle electrode. “Reprinted/adapted with permission from Ref. [196]. 2021, Elsevier”. More details on “Copyright and Licensing” are available via the following link: https://s100.copyright.com/order/91350734-cc38-4faf-b881-4a7ec6eb20a3 (accessed on 6 June 2022). (**c**) Determination of salicylic acid in cucumber leaves by interdigitated microelectrode. “Reprinted/adapted with permission from Ref. [197]. 2021, Elsevier”. More details on “Copyright and Licensing” are available via the following link: https://s100.copyright.com/order/50ecf4a3-33c1-4ddd-8c0d-471da4a1d172 (accessed on 6 June 2022). (**d**) Determination of glucose in rats by screen printing electrode. “Reprinted/adapted with permission from Ref. [198]. 2020, Elsevier”. More details on “Copyright and Licensing” are available via the following link: https://s100.copyright.com/order/6a853873-9a9f-4bcb-9e7a-885f8dc5afe6 (accessed on 6 June 2022).

However, with the development of agricultural and plant sciences, the detection of ion concentrations in crops is required, including real-time, online, in vivo, in situ, and so on. In situ detection in vivo may have better performance in studying the time response speed of plant physiology mechanism. The same is true in wearable devices, which have flourished in medicine and zoology [179,180,181,182,183]. In [184], a biosensor for human sweat and tears was developed. The study in [185] showed that a wearable electrochemical sensor based on β-CD functionalized graphene printing was prepared to monitor pH and K^+^ concentrations in sweat. Some research has examined the in situ detection, in vivo, of small organic molecules in plants [166,186,187,188,189]. The study in [165] used Au@SnO_2_-vertical graphene/Ta microelectrodes to monitor abscisic acid in cucumber in vivo. Figure 6 shows in vivo experiments of needle, interdigital, and screen-printed electrodes. It could be feasible to attain these results in crop ion detection.

In vivo detection of current technologies will inevitably elicit physiological responses in crops. Of course, if the crop is faced with other serious situations such as salt stress, the physiological response of the crop caused by the detection in the plant may be ignored. It is worth noting that plant cell injury activates the immune system by signaling through changes in Ca^2+^ concentration [190,191,192]. Electrical signals in plants are subject to external influences [193,194]. For example, electric field stimulation increases calcium influx in carrot protoplasts [195]. Some electrochemical detection methods may cause plant physiological responses to interfere with the detection results by applying currents or voltages, but these currents or voltages do not directly act on plants. Due to the complexity of organisms and individual differences, the stress response of plants is still being studied, so the in vivo detection of ASSISE still needs to continue to develop. At present, the main suggestion is to try to eliminate the physiological changes (Ca^2+^ signal transmission, etc.) caused by plant damage by extending the detection time and to eliminate the influence of voltage or current by setting comparison experiments.

### 4.3. Interference in the Detect Environment

Although the corresponding ASSISE can detect the concentrations of relevant ions, the accuracy of its detection is related to the ion activity, temperature, pH, dissolved oxygen of detection solution, the type and concentration of interfering ions, and so on. Under normal circumstances, the components of the solution to be tested, whether it is culture medium or plant body fluid, are complex, and there may be significant interference in the detection process.

The traditional method to deal with interion interference has been a chemical method, which means to remove the interfering ions in the solution by complexation, precipitation, or specific ion exchange resin, but this process is complicated and tedious. At present, the typical method integrates modern computer science to establish an ASSISE multi-array sensor for the real-time acquisition of various data in the liquid to be tested and based on the developed algorithm to eliminate the interference. As shown in [199], an algorithm was developed to eliminate the interference of pH, ionic strength, and the major ions in natural water. This paper [200] also introduced a multi-array method to detect ions in water samples. According to the study in [201], the feasibility of a multi-array ISE sensor system as a tool for urine ion composition analysis was studied.

In the past, a review was invited to explain the presence of interference in ISEs from a medical perspective. The study in [202] highlighted the effects of catheter and cannula carryover, surfactants, protein deposition, and therapeutic compounds (such as ascorbic acid) on ISEs. Moreover, the interference of Cl^−^, K^+^, Na^+^, Ca^2+^, Mg^2+^, and Li^+^ in the medical laboratory was introduced and revealed that the algorithm has been applied earlier for the correction of interference.

The data simultaneously detected by multi-array sensors were considered as a method to build an anti-interference model to improve accuracy. This method could be used in the calibration cross-reactivity among ions, pH, and temperature, etc. According to the study in [203], a multi-emission fluorescence sensor array was designed to obtain multi-dimensional data at the same time, which improved the detection efficiency and accuracy of various heavy metal ions. According to the study in [204], an SC ISE array was developed to detect vegetables.

## 5. Conclusions

Research into agricultural ion detection is a critical field of study. As we have shown in this paper, the detection research into inorganic ions is relatively mature, but the method is difficult, and there is a lack of portable miniaturized detection methods and equipment. Furthermore, the electrochemical method has great interference in the detection process due to factors such as materials, environments, and detection fluid complexity. Detection results have often been accompanied by instability. With the development of modern computer science, computer model building and algorithm analysis have shown potential in ion detections. ASSISEs are an effective means to solve this problem in the future, but most research on ASSISEs is currently focused on biomedical and environmental detection. For miniaturized ASSISEs, references include the detection of biological and organic molecules, and there have been many new achievements in these areas.

There are many other electrochemical methods for ion detection, but they have not been systematically summarized. Ion detection has significant potential in crop detection. This review considered the progress, to date, in crop detection from both in vivo and in vitro aspects. Different ion–electron transduction layers with good performance, ion selection methods, and sensor miniaturization technology should be examined for their feasibility in agricultural ASSISEs. Future research should assess whether ASSISEs could have better performance in terms of anti-interference detection, detection range, and service life as compared to other solutions.

## Figures and Tables

**Table 1:** List of potassium ISE research status.

Number	Types of Electrode	Specific Recognition Material	Transduction Layer	Response to Slope	Detection Limit	Reference
1	Glassy carbon electrode	Valinomycin	Polydioxythiophene layer	58.8 ± 0.5 mV/decade	10^−6^ M	[75]
2	Glassy carbon electrode	Valinomycin	MoO_2_ microspheres	55 mV/decade	10^−5.5^ M	[76]
3	Glassy carbon electrode	Valinomycin	Chemically reduced graphene oxide	58.4 mV/decade	10^−6.2^ M	[77]
4	Glassy carbon electrode	Valinomycin	Monolayer-protected Au clusters	57.4 mV/decade	10^−6.1^ M	[78]
5	Glassy carbon electrode	Valinomycin	A conjugated redox polymer with hydroquinone pendant groups covalently attached to the poly(3,4-ethylenedioxythiophene)	60.8 ± 0.1 and 60.9 ± 0.1 mV/decade	10^−6.7^ M	[79]
6	Paper-based electrode	Valinomycin		The fitting curve is not straight		[80]
7	Platinum microelectrode	1,3-(di-4-oxabutanol)-calix [4]arene-crown-5	The internal solid contact based on electropolymerized polypyrrole films, doped with cobaltabis(dicarbollide) ions ([3,3′-Co(1,2- C2B9H11)2]).	51 ± 2 mV/decade	10^−5.75^ M	[81]
8	Thermoplastic electrodes were fabricated using mixture of polystyrene and polycaprolactone and two types of graphite (Nano19)	Valinomycin	Carbon black	59.3 ± 1.01 mV/decade	10^−4^ M	[82]
9	Thermoplastic electrodes were fabricated using mixture of polystyrene and polycaprolactone and two types of graphite (MG1599)	Valinomycin	Carbon black	56.8 ± 1.7 mV/decade	10^−4^ M	[82]

**Table 2:** List of calcium ISE research status.

Number	Types of Electrode	Specific Recognition Material	Transduction Layer	Response to Slope	Detection Limit	Reference
1	Glassy carbon electrode	N,N-dicyclohexyl-N′,N′-dioctadecyl-diglycolic diamide (ETH 5234)	inorganic redox buffer-Ag@AgCl/1-tetradecyl-3-methylimidazolium chloride	28.3 mV/decade	10^−6.5^ M	[83]
2	Glassy carbon electrode	N,N,N′,N′-tetracyclohexyl-3-oxapentanediamide(ETH129)	Reduced graphene oxide films	29.5 mV/decade	10^−6.2^ M	[84]
3	Glass carbon electrode	ETH 5234	Reduced graphene oxide-coated black phosphorus	28.3 mV/decade	10^−5.1^ M	[85]
4	Printing electrode	Calcium ionophore II (ETH129)	Reduced graphene oxide aerogel	28.4 mV/decade	10^−6.73^ M	[86]
5	Gold electrode	Commercial calcium Ionophore II (ETH129)	N-phenyl-ethylenediamine- methacrylamide	30.2 ± 0.5 mV/decade	10^−5.5^ M	[87]
6	Golden disc electrode	ETH 5234	Bimetallic AuCu nanoparticles coupled with multi-walled carbon nanotubes	29 mV/decade	10^−6.2^ M	[88]
7	Screen-Printed Electrode	ETH 129	Reduced graphene oxide	29.1 mV/decade	10^−5.8^ M	[89]

**Table 3:** General crop culture fluid formula.

Name	Ca(NO_3_)_2_·4H_2_O mM	KNO_3_ mM	NH_4_H_2_PO_4_ mM	KH_2_PO_4_ mM	(NH_4_)_2_SO_4_ mM	K_2_SO_4_ mM	MgSO_4_·7H_2_O mM
Hoagland	4	6	1	/	/	/	2
The Japanese garden tries	4	8	1.33	/	/	/	2
Chrysanthemum of Yamazaki in Japan	2	8	1.33	/	/	/	2
Copper	4.5	5	/	1	/	/	3
The Dutch greenhouse	3.75	3	/	1.5	0.25	1.25	1
Universal trace element formula	EDTA-NaFe mM	H_3_BO_3_ mM	ZnSO_4_·7H_2_O mM	CuSO_4_·7H_2_O mM	MnSO_4_·4H_2_O mM	(NH_4_)_6_Mo_7_O_24_· 4H_2_O mM	/
/	0.071	0.046	0.000765	0.0005	0.01	0.000016	/

## Data Availability

Not applicable.

## Nomenclature

ASSISE	all-solid-state ion-selective electrode
CNT	carbon nanotube
CP	conducting polymer
DPV	differential pulse voltammetry
EIS	electrochemical impedance spectroscopy
ISE	ion-selective electrode
IUPAC	the International Union of Pure Theoretical and Applied Chemistry
MWCNT	multi-walled carbon nanotube
SC	solid contact

## References

[1] Wu Q., Bi H.-M., Han X.-J. (2021). Research Progress of Electrochemical Detection of Heavy Metal Ions. Chin. J. Anal. Chem..

[2] Mei C.J., Ahmad S.A.A. (2021). A review on the determination heavy metals ions using calixarene-based electrochemical sensors. Arab. J. Chem..

[3] Cui J., Tcherkez G. (2021). Potassium dependency of enzymes in plant primary metabolism. Plant Physiol. Biochem..

[4] Siregar A.Y., Sartsanga C., Arifudin F.S., Phengchat R., Salamah A., Ohmido N., Fukui K., Dwiranti A. (2021). Calcium ion significance on the maintenance of barley (*Hordeum vulgare*) chromosome compaction. Micron.

[5] Brownlee C. (2002). Plant K^+^ Transport: Not Just an Uphill Struggle. Curr. Biol..

[6] Noman M., Aysha J., Ketehouli T., Yang J., Du L., Wang F., Li H. (2021). Calmodulin binding transcription activators: An interplay between calcium signalling and plant stress tolerance. J. Plant Physiol..

[7] Manishankar P., Wang N., Koster P., Alatar A.A., Kudla J. (2018). Calcium Signaling during Salt Stress and in the Regulation of Ion Homeostasis. J. Exp. Bot..

[8] Guo W., Nazim H., Liang Z., Yang D. (2016). Magnesium deficiency in plants: An urgent problem. Crop J..

[9] Huang J., Wang C., Qi L., Zhang X., Tang G., Li L., Guo J., Jia Y., Dou X., Lu M. (2020). Phosphorus is more effective than nitrogen in restoring plant communities of heavy metals polluted soils. Environ. Pollut..

[10] Feng Z., Ji S., Ping J., Cui D. (2021). Recent advances in metabolomics for studying heavy metal stress in plants. TrAC Trends Anal. Chem..

[11] Anjum S.A., Tanveer M., Hussain S., Shahzad B., Ashraf U., Fahad S., Hassan W., Jan S., Khan I., Saleem M.F. (2016). Osmoregulation and antioxidant production in maize under combined cadmium and arsenic stress. Environ. Sci. Pollut. Res. Int..

[12] Nanda R., Agrawal V. (2016). Elucidation of zinc and copper induced oxidative stress, DNA damage and activation of defence system during seed germination in Cassia angustifolia Vahl. Environ. Exp. Bot..

[13] Bucker-Neto L., Paiva A.L.S., Machado R.D., Arenhart R.A., Margis-Pinheiro M. (2017). Interactions between plant hormones and heavy metals responses. Genet. Mol. Biol..

[14] Keisham M., Mukherjee S., Bhatla S.C. (2018). Mechanisms of Sodium Transport in Plants-Progresses and Challenges. Int. J. Mol. Sci..

[15] Deinlein U., Stephan A.B., Horie T., Luo W., Xu G., Schroeder J.I. (2014). Plant salt-tolerance mechanisms. Trends Plant Sci..

[16] Loutfy N., Sakuma Y., Gupta D.K., Inouhe M. (2020). Modifications of water status, growth rate and antioxidant system in two wheat cultivars as affected by salinity stress and salicylic acid. J. Plant Res..

[17] Zhu J.K. (2002). Salt and drought stress signal transduction in plants. Annu. Rev. Plant Biol..

[18] Falakboland Z., Zhou M., Zeng F., Kiani-Pouya A., Shabala L., Shabala S. (2017). Plant ionic relation and whole-plant physiological responses to waterlogging, salinity and their combination in barley. Funct. Plant Biol..

[19] Wang N., Zang J., Guo X., Wang H., Huang N., Zhao C., Zhao X., Liu J. (2022). Role of rice cultivation on fluorine distribution behavior in soda saline-alkali land. Sci. Total Environ..

[20] Shen Y., Zhu B. (2022). Effects of nitrogen and phosphorus enrichment on soil N2O emission from natural ecosystems: A global meta-analysis. Environ. Pollut..

[21] Islam S., Zhang J., Zhao Y., She M., Ma W. (2021). Genetic regulation of the traits contributing to wheat nitrogen use efficiency. Plant Sci..

[22] Hasan A., Nanakali N.M.Q., Salihi A., Rasti B., Sharifi M., Attar F., Derakhshankhah H., Mustafa I.A., Abdulqadir S.Z., Falahati M. (2020). Nanozyme-based sensing platforms for detection of toxic mercury ions: An alternative approach to conventional methods. Talanta.

[23] Buchberger W.W. (2000). Detection techniques in ion analysis: What are our choices?. J. Chromatogr. A.

[24] Suman S., Singh R. (2019). Anion selective electrodes: A brief compilation. Microchem. J..

[25] Shao Y., Ying Y., Ping J. (2020). Recent advances in solid-contact ion-selective electrodes: Functional materials, transduction mechanisms, and development trends. Chem. Soc. Rev..

[26] Consalteri A., Rigato A., Clamor L., Giandon P. (1992). Determination of Nitrate in Vegetables Using an Ion-Selective Electrode. J. Food Compos. Anal..

[27] Lindner E., Pendley B.D. (2013). A tutorial on the application of ion-selective electrode potentiometry: An analytical method with unique qualities, unexplored opportunities and potential pitfalls; tutorial. Anal. Chim. Acta.

[28] Pietrzak K., Krstulovic N., Blazeka D., Car J., Malinowski S., Wardak C. (2022). Metal oxide nanoparticles as solid contact in ion-selective electrodes sensitive to potassium ions. Talanta.

[29] Tsuchiya K., Akatsuka T., Abe Y., Komaba S. (2021). Design of all-solid-state chloride and nitrate ion-selective electrodes using anion insertion materials of electrodeposited poly(allylamine)-MnO_2_ composite. Electrochim. Acta.

[30] Mazzaracchio V., Serani A., Fiore L., Moscone D., Arduini F. (2021). All-solid state ion-selective carbon black-modified printed electrode for sodium detection in sweat. Electrochim. Acta.

[31] Ronkainen N.J., Halsall H.B., Heineman W.R. (2010). Electrochemical biosensors. Chem. Soc. Rev..

[32] Kamyabi M.A., Kazemi D., Bikas R., Soleymani-Bonoti F. (2021). Investigation of the Hg(II) biosorption from wastewater by using garlic plant and differential pulse voltammetry. Anal. Biochem..

[33] Salaria K., Mehta N., Krishna C., Mehta S.K. (2021). Electrochemical detection of TNT using CdS nanoparticles via cyclic voltammetry and amperometry. Curr. Res. Green Sustain. Chem..

[34] Patella B., Aiello G., Drago G., Torino C., Vilasi A., O’Riordan A., Inguanta R. (2022). Electrochemical detection of chloride ions using Ag-based electrodes obtained from compact disc. Anal. Chim. Acta.

[35] Manjakkal L., Djurdjic E., Cvejin K., Kulawik J., Zaraska K., Szwagierczak D. (2015). Electrochemical Impedance Spectroscopic Analysis of RuO_2_ Based Thick Film pH Sensors. Electrochim. Acta.

[36] Xu B., Zhang W.-D. (2010). Modification of vertically aligned carbon nanotubes with RuO_2_ for a solid-state pH sensor. Electrochim. Acta.

[37] Manjakkal L., Cvejin K., Kulawik J., Zaraska K., Socha R.P., Szwagierczak D. (2016). X-ray photoelectron spectroscopic and electrochemical impedance spectroscopic analysis of RuO_2_-Ta_2_O_5_ thick film pH sensors. Anal. Chim. Acta.

[38] Cheong Y.H., Ge L., Lisak G. (2021). Highly reproducible solid contact ion selective electrodes: Emerging opportunities for potentiometry—A review. Anal. Chim. Acta.

[39] Abdel-Haleem F.M., Saad M., Barhoum A., Bechelany M., Rizk M.S. (2018). PVC membrane, coated-wire, and carbon-paste ion-selective electrodes for potentiometric determination of galantamine hydrobromide in physiological fluids. Mater. Sci. Eng. C Mater. Biol. Appl..

[40] Bobacka J. (2006). Conducting Polymer-Based Solid-State Ion-Selective Electrodes. Electroanalysis.

[41] Hu J., Stein A., Bühlmann P. (2016). Rational design of all-solid-state ion-selective electrodes and reference electrodes. TrAC Trends Anal. Chem..

[42] Liu Q., Wang F., Qiao Y., Zhang S., Ye B. (2010). Polyaniline Langmuir–Blodgett film modified glassy carbon electrode as a voltammetric sensor for determination of Ag+ ions. Electrochim. Acta.

[43] Pięk M., Piech R., Paczosa-Bator B. (2016). All-solid-state nitrate selective electrode with graphene/tetrathiafulvalene nanocomposite as high redox and double layer capacitance solid contact. Electrochim. Acta.

[44] Wardak C. (2014). Solid contact Zn^2+^ -selective electrode with low detection limit and stable and reversible potential. Open Chem..

[45] Jiang W., Liu C., Zhao Y., Waterhouse G.I., Zhang Z., Yu L. (2019). A solid-contact Pb^2+^-selective electrode based on a hydrophobic polyaniline microfiber film as the ion-to-electron transducer. Synth. Met..

[46] Pietrzak K., Wardak C. (2021). Comparative study of nitrate all solid state ion-selective electrode based on multiwalled carbon nanotubes-ionic liquid nanocomposite. Sens. Actuators B Chem..

[47] Abdollahzadeh M., Bayatsarmadi B., Vepsäläinen M., Razmjou A., Asadnia M. (2022). Highly stable Li+ selective electrode with metal-organic framework as ion-to-electron transducer. Sens. Actuators B Chem..

[48] Wardak C. (2015). Solid contact cadmium ion-selective electrode based on ionic liquid and carbon nanotubes. Sens. Actuators B Chem..

[49] Zeng X., Liu Y., Jiang X., Waterhouse G.I., Zhang Z., Yu L. (2021). Improving the stability of Pb^2+^ ion-selective electrodes by using 3D polyaniline nanowire arrays as the inner solid-contact transducer. Electrochim. Acta.

[50] Li J., Qin W. (2019). A freestanding all-solid-state polymeric membrane Cu^2+^-selective electrode based on three-dimensional graphene sponge. Anal. Chim. Acta.

[51] Sharma R., Geranpayehvaghei M., Ejeian F., Razmjou A., Asadnia M. (2021). Recent advances in polymeric nanostructured ion selective membranes for biomedical applications. Talanta.

[52] Ammann D., Chao P., Simon W. (1987). Valinomycin-based K^+^ selective microelectrodes with low electrical membrane resistance. Neurosci. Lett..

[53] Vuilleumier P., Gazzotti P., Carafoli E., Simon W. (1977). The translocation of Ca^2+^ across phospholipid bilayers induced by a synthetic neutral Ca^2+^-ionophore. Biochim. Biophys. Acta (BBA)-Biomembr..

[54] Liu F., Wang M., Wang X., Wang P., Shen W., Ding S., Wang Y. (2019). Fabrication and application of nanoporous polymer ion-track membranes. Nanotechnology.

[55] Wang S., Zhong L., Gan S., Tang Y., Qiu S., Lyu Y., Ma Y., Niu L. (2021). Defective vs high-quality graphene for solid-contact ion-selective electrodes: Effects of capacitance and hydrophobicity. Electrochem. Commun..

[56] Zeng X., Yu S., Yuan Q., Qin W. (2016). Solid-contact K^+^-selective electrode based on three-dimensional molybdenum sulfide nanoflowers as ion-to-electron transducer. Sens. Actuators B Chem..

[57] Xu J., Jia F., Li F., An Q., Gan S., Zhang Q., Ivaska A., Niu L. (2016). Simple and Efficient Synthesis of Gold Nanoclusters and Their Performance as Solid Contact of Ion Selective Electrode. Electrochim. Acta.

[58] Reid P.H., York E.T. (1958). Effect of Nutrient Deficiencies on Growth and Fruiting Characteristics of Peanuts in Sand Cultures. Agron. J..

[59] Li Z. (2017). An All-Solid-State Polymeric Membrane chloride ion-selective Electrode with Nanowires poly(3,4-ethylenedioxythiophene) as Solid Contact. Int. J. Electrochem. Sci..

[60] Ko K.H., Kim G.H., Song J.G., Kim S.G. (2022). A novel cyclic voltammetric determination of free chlorine generated by ozone disinfection in seawater aquariums. Chin. J. Anal. Chem..

[61] Murata M., Ivandini T.A., Shibata M., Nomura S., Fujishima A., Einaga Y. (2008). Electrochemical detection of free chlorine at highly boron-doped diamond electrodes. J. Electroanal. Chem..

[62] Pathiratne K.A.S., Skandaraja S.S., Jayasena E.M.C.M. (2008). Linear sweep voltammetric determination of free chlorine in waters. J. Natl. Sci. Found. Sri Lanka.

[63] Bujes-Garrido J., Arcos-Martinez M.J. (2016). Disposable sensor for electrochemical determination of chloride ions. Talanta.

[64] Thiagarajan S., Wu Z.-Y., Chen S.-M. (2011). Amperometric determination of sodium hypochlorite at poly MnTAPP-nano Au film modified electrode. J. Electroanal. Chem..

[65] Kumar D.R., Kesavan S., Nguyen T.T., Hwang J., Lamiel C., Shim J.-J. (2017). Polydopamine@electrochemically reduced graphene oxide-modified electrode for electrochemical detection of free-chlorine. Sens. Actuators B Chem..

[66] Salazar P., Martín M., González-Mora J.L., González-Elipe A.R. (2016). Application of Prussian Blue electrodes for amperometric detection of free chlorine in water samples using Flow Injection Analysis. Talanta.

[67] Manjakkal L., Szwagierczak D., Dahiya R. (2020). Metal oxides based electrochemical pH sensors: Current progress and future perspectives. Prog. Mater. Sci..

[68] Sivaranjanee R., Senthil Kumar P., Saravanan R., Govarthanan M. (2022). Electrochemical sensing system for the analysis of emerging contaminants in aquatic environment: A review. Chemosphere.

[69] Brett C.M.A., Oliveira-Brett A.M. (2011). Electrochemical sensing in solution—origins, applications and future perspectives. J. Solid State Electrochem..

[70] Gemene K.L., Bakker E. (2009). Measurement of total calcium by flash chronopotentiometry at polymer membrane ion-selective electrodes. Anal. Chim. Acta.

[71] Shvarev A., Bakker E. (2004). Distinguishing free and total calcium with a single pulsed galvanostatic ion-selective electrode. Talanta.

[72] Shvarev A., Bakker E. (2003). Pulsed Galvanostatic Control of Ionophore-Based Polymeric Ion Sensors. Anal. Chem..

[73] Liu S., Ding J., Qin W. (2018). Current pulse based ion-selective electrodes for chronopotentiometric determination of calcium in seawater. Anal. Chim. Acta.

[74] Bakker E., Buhlmann P., Pretsch E. (2004). The phase-boundary potential model. Talanta.

[75] Kondratyeva Yevgeniya O., Tolstopjatova Elena G., Kirsanov Dmitry O., Mikhelson Konstantin N. (2020). Chronoamperometric and coulometric analysis with ionophore-based ion-selective electrodes: A modified theory and the potassium ion assay in serum samples. Sens. Actuators B Chem..

[76] Zeng X., Qin W. (2017). A solid-contact potassium-selective electrode with MoO_2_ microspheres as ion-to-electron transducer. Anal. Chim. Acta.

[77] Ping J., Wang Y., Wu J., Ying Y. (2011). Development of an all-solid-state potassium ion-selective electrode using graphene as the solid-contact transducer. Electrochem. Commun..

[78] An Q., Jiao L., Jia F., Ye J., Li F., Gan S., Zhang Q., Ivaska A., Niu L. (2016). Robust single-piece all-solid-state potassium-selective electrode with monolayer-protected Au clusters. J. Electroanal. Chem..

[79] Ivanko I., Lindfors T., Emanuelsson R., Sjödin M. (2021). Conjugated redox polymer with poly(3,4-ethylenedioxythiophene) backbone and hydroquinone pendant groups as the solid contact in potassium-selective electrodes. Sens. Actuators B Chem..

[80] Kassal P., Sigurnjak M., Steinberg I.M. (2019). Paper-based ion-selective optodes for continuous sensing: Reversible potassium ion monitoring. Talanta.

[81] Zine N., Bausells J., Vocanson F., Lamartine R., Asfari Z., Teixidor F., Crespo E., de Oliveira I.M., Samitier J., Errachid A. (2006). Potassium-ion selective solid contact microelectrode based on a novel 1,3-(di-4-oxabutanol)-calix[4]arene-crown-5 neutral carrier. Electrochim. Acta.

[82] Ozer T., Henry C.S. (2022). All-solid-state potassium-selective sensor based on carbon black modified thermoplastic electrode. Electrochim. Acta.

[83] Zeng X., Qin W. (2020). A solid-contact Ca^2+^-selective electrode based on an inorganic redox buffer of Ag@AgCl/1-tetradecyl-3-methylimidazolium chloride as ion-to-electron transducer. Talanta.

[84] Hernández R., Riu J., Bobacka J., Vallés C., Jiménez P., Benito A.M., Maser W.K., Rius F.X. (2012). Reduced Graphene Oxide Films as Solid Transducers in Potentiometric All-Solid-State Ion-Selective Electrodes. J. Phys. Chem. C.

[85] Yang Q. (2019). All-solid-state Ca^2+^ Ion-selective Electrode with Black Phosphorus and Reduced Graphene Oxide as the Mediator Layer. Int. J. Electrochem. Sci..

[86] Kim M.-Y., Lee J.-W., Park D.J., Lee J.-Y., Myung N.V., Kwon S.H., Lee K.H. (2021). Highly stable potentiometric sensor with reduced graphene oxide aerogel as a solid contact for detection of nitrate and calcium ions. J. Electroanal. Chem..

[87] Abramova N., Moral-Vico J., Soley J., Ocana C., Bratov A. (2016). Solid contact ion sensor with conducting polymer layer copolymerized with the ion-selective membrane for determination of calcium in blood serum. Anal. Chim. Acta.

[88] Liu Y., Liu Y., Yan R., Gao Y., Wang P. (2020). Bimetallic AuCu nanoparticles coupled with multi-walled carbon nanotubes as ion-to-electron transducers in solid-contact potentiometric sensors. Electrochim. Acta.

[89] Ping J., Wang Y., Ying Y., Wu J. (2012). Application of electrochemically reduced graphene oxide on screen-printed ion-selective electrode. Anal. Chem..

[90] Volf M.R., Batista-Silva W., Silvério A.D., dos Santos L.G., Tiritan C.S. (2022). Effect of potassium fertilization in sandy soil on the content of essential nutrients in soybean leaves. Ann. Agric. Sci..

[91] Buoso S., Musetti R., Marroni F., Calderan A., Schmidt W., Santi S. (2022). Infection by phloem-limited phytoplasma affects mineral nutrient homeostasis in tomato leaf tissues. J. Plant Physiol..

[92] Paz-Ares J., Puga M.I., Rojas-Triana M., Martinez-Hevia I., Diaz S., Poza-Carrion C., Minambres M., Leyva A. (2022). Plant adaptation to low phosphorus availability: Core signaling, crosstalks, and applied implications. Mol. Plant.

[93] Wang P., Limpens E., Yao R. (2022). Orchestrating plant direct and indirect phosphate uptake pathways. Trends Plant Sci..

[94] Xiao J., Dong S., Shen H., Li S., Zhi Y., Mu Z., Ding C. (2022). Phosphorus addition promotes Nitrogen retention in alpine grassland plants while increasing N deposition. Catena.

[95] Xu K., Wu B., Wan J., Li Y., Li M. (2022). A potentiometric phosphate ion sensor based on electrochemically modified nickel electrode. Electrochim. Acta.

[96] Sartori M.C., Zezza T.R., Paim L.L., Stradiotto N.R. (2014). Potentiometric determination of phosphorus in biodiesel using chemically modified electrode with cobalt film. Fuel.

[97] Marcinek S., Chapoulie A., Salaun P., Smith S., Omanovic D. (2021). Revised application of copper ion selective electrode (Cu-ISE) in marine waters: A new meta-calibration approach. Talanta.

[98] Birinci A., Eren H., Coldur F., Coskun E., Andac M. (2016). Rapid determination of trace level copper in tea infusion samples by solid contact ion selective electrode. J. Food Drug Anal..

[99] Ali Tamer A.w.a.d., Farag Ahmed A., Mohamed Gehad G. (2014). Potentiometric determination of iron in polluted water samples using new modified Fe(III)-screen printed ion selective electrode. J. Ind. Eng. Chem..

[100] Park S., Maier C.S., Koley D. (2021). Anodic Stripping Voltammetry on a Carbon-based Ion-Selective Electrode. Electrochim. Acta.

[101] Makarychev-Mikhailov S., Shvarev A.A., Bakker E. (2004). Pulstrodes: Triple Pulse Control of Potentiometric Sensors. J. Am. Chem. Soc..

[102] Peshkova M.A., Mikhel’son K.N. (2010). Ion-selective electrodes under galvanostatic polarization conditions. Russ. J. Electrochem..

[103] Lisak G., Sokalski T., Bobacka J., Harju L., Mikhelson K., Lewenstam A. (2011). Tuned galvanostatic polarization of solid-state lead-selective electrodes for lowering of the detection limit. Anal. Chim. Acta.

[104] (2021). Agricultural Fertilizer Application Amount. https://data.stats.gov.cn/easyquery.htm?cn=C01.

[105] Xu F. (2021). Research on Key Sensing Technology of Available Nutrients in Hydroponic Solution for Plant Factory. Ph.D. Thesis.

[106] Lopes V., De Carvalho A.A., Bertolucci S.K.V., Roza H.L.H., Figueiredo F.C., Pinto J.E.B.P. (2019). Macronutrient Suppression in Nutrient Solution Alters the Growth and Citral Content of Cymbopogon flexuosus. J. Agric. Sci..

[107] Alvarenga I., Boldrin P., Pacheco F.V., Silva S.T., Bertolucci S.K.V., Pinto J.E.B.P. (2015). Effects on growth, essential oil content and composition of the volatile fraction of *Achillea millefolium* L. cultivated in hydroponic systems deficient in macro- and microelements. Sci. Hortic..

[108] Silva T.C., Bertolucci S.K., Carvalho A.A., Tostes W.N., Alvarenga I.C., Pacheco F.V., de Assis R.M., Honorato A.D.C., Pinto J.E. (2021). Macroelement omission in hydroponic systems changes plant growth and chemical composition of *Melissa officinalis* L. essential oil. J. Appl. Res. Med. Aromat. Plants.

[109] Cho W.-J., Kim H.-J., Jung D.-H., Kim D.-W., Ahn T.I., Son J.E. (2018). On-site ion monitoring system for precision hydroponic nutrient management. Comput. Electron. Agric..

[110] Griffiths M., Roy S., Guo H., Seethepalli A., Huhman D., Ge Y., Sharp R.E., Fritschi F.B., York L.M. (2021). A multiple ion-uptake phenotyping platform reveals shared mechanisms affecting nutrient uptake by roots. Plant Physiol..

[111] Jung D.-H., Kim H.-J., Cho W.-J., Park S.H., Yang S.-H. (2019). Validation testing of an ion-specific sensing and control system for precision hydroponic macronutrient management. Comput. Electron. Agric..

[112] Hoang N.N., Kitaya Y., Shibuya T., Endo R. (2019). Development of an in vitro hydroponic culture system for wasabi nursery plant production—Effects of nutrient concentration and supporting material on plantlet growth. Sci. Hortic..

[113] De Paula B.V., Marques A.C.R., Rodrigues L.A.T., de Souza R.O.S., Kulmann M.S.D.S., Kaminski J., Ceretta C.A., de Melo G.W.B., Mayer N.A., Antunes L.E. (2018). Morphological and kinetic parameters of the uptake of nitrogen forms in clonal peach rootstocks. Sci. Hortic..

[114] Wen Y., Mao Y., Kang Z., Luo Q. (2019). Application of an ammonium ion-selective electrode for the real-time measurement of ammonia nitrogen based on pH and temperature compensation. Measurement.

[115] Huang Y., Li J., Yin T., Jia J., Ding Q., Zheng H., Chen C.-T.A., Ye Y. (2015). A novel all-solid-state ammonium electrode with polyaniline and copolymer of aniline/2,5-dimethoxyaniline as transducers. J. Electroanal. Chem..

[116] Wang H., Yuan B., Yin T., Qin W. (2020). Alternative coulometric signal readout based on a solid-contact ion-selective electrode for detection of nitrate. Anal. Chim. Acta.

[117] Liu Y., Xue Y., Xie B., Zhu S., Lu X., Liang C., Tian J. (2020). Complex gene regulation between young and old soybean leaves in responses to manganese toxicity. Plant Physiol. Biochem..

[118] Ari B., Oz E., Can S.Z., Bakirdere S. (2022). Bioaccessibility and bioavailability of selenium species in Se-enriched leeks (Allium Porrum) cultivated by hydroponically. Food Chem..

[119] Tan Z., Wu W., Yin N., Jia M., Chen X., Bai Y., Wu H., Zhang Z., Li P. (2020). Determination of selenium in food and environmental samples using a gold nanocages/fluorinated graphene nanocomposite modified electrode. J. Food Compos. Anal..

[120] Sharma V.K., McDonald T.J., Sohn M., Anquandah G.A.K., Pettine M., Zboril R. (2017). Assessment of toxicity of selenium and cadmium selenium quantum dots: A review. Chemosphere.

[121] Lenz M., Lens P.N. (2009). The essential toxin: The changing perception of selenium in environmental sciences. Sci. Total Environ..

[122] Jain R., Thakur A., Kumar P., Pooja D. (2018). Au/ZnO nanocomposites decorated ITO electrodes for voltammetric sensing of selenium in water. Electrochim. Acta.

[123] Wu Y., Li X., Yu L., Wang T., Wang J., Liu T. (2022). Review of soil heavy metal pollution in China: Spatial distribution, primary sources, and remediation alternatives. Resour. Conserv. Recycl..

[124] Mishra V.K., Upadhyaya A.R., Pandey S.K., Tripathi B.D. (2008). Heavy metal pollution induced due to coal mining effluent on surrounding aquatic ecosystem and its management through naturally occurring aquatic macrophytes. Bioresour. Technol..

[125] Kadim M.K., Risjani Y. (2022). Biomarker for monitoring heavy metal pollution in aquatic environment: An overview toward molecular perspectives. Emerg. Contam..

[126] Li K., Wang J., Zhang Y. (2022). Heavy metal pollution risk of cultivated land from industrial production in China: Spatial pattern and its enlightenment. Sci. Total Environ..

[127] Wortmann C.S., Tarkalson D.D., Shapiro C.A., Dobermann A.R., Ferguson R.B., Hergert G.W., Walters D. (2011). Nitrogen Use Efficiency of Irrigated Corn for Three Cropping Systems in Nebraska. Agron. J..

[128] Hawkesford M.J. (2017). Genetic variation in traits for nitrogen use efficiency in wheat. J. Exp. Bot..

[129] Xiao D., Vu Q.H., Le B.T. (2021). Salt content in saline-alkali soil detection using visible-near infrared spectroscopy and a 2D deep learning. Microchem. J..

[130] Wei B., Yang L. (2010). A review of heavy metal contaminations in urban soils, urban road dusts and agricultural soils from China. Microchem. J..

[131] Dong W., Zhang Y., Quan X. (2020). Health risk assessment of heavy metals and pesticides: A case study in the main drinking water source in Dalian, China. Chemosphere.

[132] Nguyen L.D., Doan T.C.D., Huynh T.M., Nguyen V.N.P., Dinh H.H., Dang D.M.T., Dang C.M. (2021). An electrochemical sensor based on polyvinyl alcohol/chitosan-thermally reduced graphene composite modified glassy carbon electrode for sensitive voltammetric detection of lead. Sens. Actuators B Chem..

[133] Martin-Yerga D., Alvarez-Martos I., Blanco-Lopez M.C., Henry C.S., Fernandez-Abedul M.T. (2017). Point-of-need simultaneous electrochemical detection of lead and cadmium using low-cost stencil-printed transparency electrodes. Anal. Chim. Acta.

[134] Mendes Ana Luiza G., Nascimento Mariele S., Picoloto Rochele S., Flores Erico M.M., Mello Paola A. (2020). A sample preparation method for fluoride detection by potentiometry with ion-selective electrode in medicinal plants. J. Fluor. Chem..

[135] Martín-Yerga D., Álvarez-Martos I., Blanco-López M.C., Henry C.S., Fernández-Abedul M.T. (2021). Rapid potentiometric sensor for determination of Cu(II) ions in food samples. Microchem. J..

[136] Bhanot V., Fadanavis S.V., Panwar J. (2021). Revisiting the architecture, biosynthesis and functional aspects of the plant cuticle: There is more scope. Environ. Exp. Bot..

[137] Yeats T.H., Rose J.K. (2013). The formation and function of plant cuticles. Plant Physiol..

[138] Krikstolaityte V., Ding R., Xia E.C.H., Lisak G. (2020). Paper as sampling substrates and all-integrating platforms in potentiometric ion determination. TrAC Trends Anal. Chem..

[139] Fernández-La-Villa A., Pozo-Ayuso D.F., Castaño-Álvarez M. (2019). Microfluidics and electrochemistry: An emerging tandem for next-generation analytical microsystems. Curr. Opin. Electrochem..

[140] Afsarimanesh N., Nag A., Alahi E.E., Han T., Mukhopadhyay S. (2020). Interdigital sensors: Biomedical, environmental and industrial applications. Sens. Actuators A Phys..

[141] Nery E.W., Kubota L.T. (2013). Sensing approaches on paper-based devices: A review. Anal. Bioanal. Chem..

[142] Noviana E., McCord C.P., Clark K.M., Jang I., Henry C.S. (2020). Electrochemical paper-based devices: Sensing approaches and progress toward practical applications. Lab Chip.

[143] Arduini F., Cinti S., Scognamiglio V., Moscone D., Palleschi G. (2017). How cutting-edge technologies impact the design of electrochemical (bio)sensors for environmental analysis. A review. Anal. Chim. Acta.

[144] Moya A., Gabriel G., Villa R., del Campo F.J. (2017). Inkjet-printed electrochemical sensors. Curr. Opin. Electrochem..

[145] Goh G.L., Agarwala S., Tan Y.J., Yeong W.Y. (2018). A low cost and flexible carbon nanotube pH sensor fabricated using aerosol jet technology for live cell applications. Sens. Actuators B Chem..

[146] Channon R.B., Nguyen M.P., Scorzelli A.G., Henry E.M., Volckens J., Dandy D.S., Henry C.S. (2018). Rapid flow in multilayer microfluidic paper-based analytical devices. Lab Chip.

[147] Liu F., Zhang C. (2015). A novel paper-based microfluidic enhanced chemiluminescence biosensor for facile, reliable and highly-sensitive gene detection of Listeria monocytogenes. Sens. Actuators B Chem..

[148] De Araujo W.R., Frasson C.M.R., Ameku W.A., Silva J.R., Angnes L., Paixao T. (2017). Single-Step Reagentless Laser Scribing Fabrication of Electrochemical Paper-Based Analytical Devices. Angew. Chem. Int. Ed. Engl..

[149] Dias Anderson A., Cardoso Thiago M.G., Chagas Cyro L.S., Oliveira Virgílio X.G., Munoz Rodrigo A.A., Henry Charles S., Santana Mário H.P., Paixão Thiago R.L.C., Coltro Wendell K.T. (2018). Detection of Analgesics and Sedation Drugs in Whiskey Using Electrochemical Paper-based Analytical Devices. Electroanalysis.

[150] Oliveira V.X.G., Dias A.A., Carvalho L.L., Cardoso T.M.G., Colmati F., Coltro W.K.T. (2018). Determination of Ascorbic Acid in Commercial Tablets Using Pencil Drawn Electrochemical Paper-based Analytical Devices. Anal. Sci..

[151] Parrilla M., Canovas R., Andrade F.J. (2017). Paper-based enzymatic electrode with enhanced potentiometric response for monitoring glucose in biological fluids. Biosens. Bioelectron..

[152] Nunez-Bajo E., Blanco-Lopez M., Costa-García A., Fernández-Abedul M. (2017). Gold Nanostructuration in Paper-based Electrodes. Procedia Technol..

[153] Carvalhal R.F., Kfouri M.S., Piazetta M.H.D.O., Gobbi A.L., Kubota L.T. (2010). Electrochemical Detection in a Paper-Based Separation Device. Anal. Chem..

[154] Firdaus C., Rizam M., Rusop M., Hidayah S. (2012). Characterization of ZnO and ZnO: TiO_2_ Thin Films Prepared by Sol-Gel Spray-Spin Coating Technique. Procedia Eng..

[155] Kit-Anan W., Olarnwanich A., Sriprachuabwong C., Karuwan C., Tuantranont A., Wisitsoraat A., Srituravanich W., Pimpin A. (2012). Disposable paper-based electrochemical sensor utilizing inkjet-printed Polyaniline modified screen-printed carbon electrode for Ascorbic acid detection. J. Electroanal. Chem..

[156] Ambaye A.D., Kefeni K.K., Mishra S.B., Nxumalo E.N., Ntsendwana B. (2021). Recent developments in nanotechnology-based printing electrode systems for electrochemical sensors. Talanta.

[157] Li M., Li Y.T., Li D.W., Long Y.T. (2012). Recent developments and applications of screen-printed electrodes in environmental assays—A review. Anal. Chim. Acta.

[158] Trojanowicz M. (2016). Impact of nanotechnology on design of advanced screen-printed electrodes for different analytical applications. TrAC Trends Anal. Chem..

[159] Cheong Y.H., Ge L., Zhao N., Teh L.K., Lisak G. (2020). Ion selective electrodes utilizing a ferrocyanide doped redox active screen-printed solid contact—Impact of electrode response to conditioning. J. Electroanal. Chem..

[160] Xing G., Luo B., Qin J., Wang X., Hou P., Zhang H., Wang C., Wang J., Li A. (2021). A probe-free electrochemical immunosensor for methyl jasmonate based on ferrocene functionalized-carboxylated graphene-multi-walled carbon nanotube nanocomposites. Talanta.

[161] Gao J., Huang W., Chen Z., Yi C., Jiang L. (2019). Simultaneous detection of glucose, uric acid and cholesterol using flexible microneedle electrode array-based biosensor and multi-channel portable electrochemical analyzer. Sens. Actuators B Chem..

[162] Sharma S., Takagi E., Cass T., Tsugawa W., Sode K. (2017). Minimally Invasive Microneedle Array Electrodes Employing Direct Electron Transfer Type Glucose Dehydrogenase for the Development of Continuous Glucose Monitoring Sensors. Procedia Technol..

[163] Caliò A., Dardano P., Di Palma V., Bevilacqua M.F., Di Matteo A., Iuele H., De Stefano L. (2016). Polymeric microneedles based enzymatic electrodes for electrochemical biosensing of glucose and lactic acid. Sens. Actuators B Chem..

[164] Jeon E., Choi S., Yeo K.-H., Park K.S., Rathod M.L., Lee J. (2017). Development of electrical conductivity measurement technology for key plant physiological information using microneedle sensor. J. Micromech. Microeng..

[165] Wang Z., Xue L., Li M., Li C., Li P., Li H. (2021). Au@SnO_2_-vertical graphene-based microneedle sensor for in-situ determination of abscisic acid in plants. Mater. Sci. Eng. C Mater. Biol. Appl..

[166] Li H., Wang C., Wang X., Hou P., Luo B., Song P., Pan D., Li A., Chen L. (2019). Disposable stainless steel-based electrochemical microsensor for in vivo determination of indole-3-acetic acid in soybean seedlings. Biosens. Bioelectron..

[167] Bollella P., Sharma S., Cass A.E.G., Antiochia R. (2019). Microneedle-based biosensor for minimally-invasive lactate detection. Biosens. Bioelectron..

[168] Debosz M., Wieczorek M., Paluch J., Migdalski J., Bas B., Koscielniak P. (2020). 3D-printed flow manifold based on potentiometric measurements with solid-state ion-selective electrodes and dedicated to multicomponent water analysis. Talanta.

[169] Debosz M., Kozma J., Porada R., Wieczorek M., Paluch J., Gyurcsanyi R.E., Migdalski J., Koscielniak P. (2021). 3D-printed manifold integrating solid contact ion-selective electrodes for multiplexed ion concentration measurements in urine. Talanta.

[170] Dabbagh S.R., Sarabi M.R., Rahbarghazi R., Sokullu E., Yetisen A.K., Tasoglu S. (2021). 3D-printed microneedles in biomedical applications. iScience.

[171] Rutkowska M., Lindfors T., Boeva Z., Strawski M. (2021). Low-cost flexible laminated graphene paper solid-contact ion-selective electrodes. Sens. Actuators B Chem..

[172] Lin L., Yin Y., Starostin S.A., Xu H., Li C., Wu K., He C., Hessel V. (2021). Microfluidic fabrication of fluorescent nanomaterials: A review. Chem. Eng. J..

[173] Schmidt-Speicher L.M., Länge K. (2021). Microfluidic integration for electrochemical biosensor applications. Curr. Opin. Electrochem..

[174] Shi H., Jiang S., Liu B., Liu Z., Reis N.M. (2021). Modern Microfluidic Approaches for Detection and Quantitation of Ions. Microchem. J..

[175] Ding R., Fiedoruk-Pogrebniak M., Pokrzywnicka M., Koncki R., Bobacka J., Lisak G. (2020). Solid reference electrode integrated with paper-based microfluidics for potentiometric ion sensing. Sens. Actuators B Chem..

[176] Liu J., Xu X., Wu A., Song S., Kuang H., Liu L., Wang Z., Xu L., Sun M., Xu C. (2022). An immunochromatographic assay for the rapid detection of oxadixyl in cucumber, tomato and wine samples. Food Chem..

[177] Rezaeian F.M., Zimmermann B.F. (2022). Simplified analysis of flavanols in matcha tea. Food Chem..

[178] Zhao Z., Liu J., Jia R., Bao S., Haixia, Chen X. (2019). Physiological and TMT-based proteomic analysis of oat early seedlings in response to alkali stress. J. Proteom..

[179] Zuliani C., Diamond D. (2012). Opportunities and challenges of using ion-selective electrodes in environmental monitoring and wearable sensors. Electrochim. Acta.

[180] An Q., Gan S., Xu J., Bao Y., Wu T., Kong H., Zhong L., Ma Y., Song Z., Niu L. (2019). A multichannel electrochemical all-solid-state wearable potentiometric sensor for real-time sweat ion monitoring. Electrochem. Commun..

[181] Parrilla M., Cuartero M., Crespo G.A. (2019). Wearable potentiometric ion sensors. TrAC Trends Anal. Chem..

[182] Liu H., Gu Z., Zhao Q., Li S., Ding X., Xiao X., Xiu G. (2022). Printed circuit board integrated wearable ion-selective electrode with potential treatment for highly repeatable sweat monitoring. Sens. Actuators B Chem..

[183] Yang Q., Rosati G., Abarintos V., Aroca M.A., Osma J.F., Merkoci A. (2022). Wearable and fully printed microfluidic nanosensor for sweat rate, conductivity, and copper detection with healthcare applications. Biosens. Bioelectron..

[184] Han J., Li M., Li H., Li C., Ye J., Yang B. (2020). Pt-poly(L-lactic acid) microelectrode-based microsensor for in situ glucose detection in sweat. Biosens. Bioelectron..

[185] Cui X., Bao Y., Han T., Liu Z., Ma Y., Sun Z. (2022). A wearable electrochemical sensor based on beta-CD functionalized graphene for pH and potassium ion analysis in sweat. Talanta.

[186] Coppede N., Janni M., Bettelli M., Maida C.L., Gentile F., Villani M., Ruotolo R., Iannotta S., Marmiroli N., Marmiroli M. (2017). An in vivo biosensing, biomimetic electrochemical transistor with applications in plant science and precision farming. Sci. Rep..

[187] Pandey R., Teig-Sussholz O., Schuster S., Avni A., Shacham-Diamand Y. (2018). Integrated electrochemical Chip-on-Plant functional sensor for monitoring gene expression under stress. Biosens. Bioelectron..

[188] Lew T.T.S., Koman V.B., Silmore K.S., Seo J.S., Gordiichuk P., Kwak S.Y., Park M., Ang M.C., Khong D.T., Lee M.A. (2020). Real-time detection of wound-induced H_2_O_2_ signalling waves in plants with optical nanosensors. Nat. Plants.

[189] Hu Y., Zhao J., Li H., Wang X., Hou P., Wang C., Li A., Chen L. (2018). In vivo detection of salicylic acid in sunflower seedlings under salt stress. RSC Adv..

[190] Hander T., Fernandez-Fernandez A.D., Kumpf R.P., Willems P., Schatowitz H., Rombaut D., Staes A., Nolf J., Pottie R., Yao P. (2019). Damage on plants activates Ca^2+^-dependent metacaspases for release of immunomodulatory peptides. Science.

[191] Morimoto K., van der Hoorn R.A.L. (2019). Plant Biology: Proteolytic Release of Damage Signals. Curr. Biol..

[192] Börnke F., Rocksch T. (2018). Thigmomorphogenesis—Control of plant growth by mechanical stimulation. Sci. Hortic..

[193] Lee S., Oh M.-M. (2021). Electric stimulation promotes growth, mineral uptake, and antioxidant accumulation in kale (*Brassica oleracea* var. *acephala*). Bioelectrochemistry.

[194] Fromm J., Lautner S. (2007). Electrical signals and their physiological significance in plants. Plant Cell Environ..

[195] Graziana A., Ranjeva R., Teissie J. (1990). External Electric Fields Stimulate the Electrogenic Calcium/Sodium Exchange in Plant Protoplasts. Biochemistry.

[196] Yang L., Ma Y., Ye J. (2021). In vivo detection of L-tryptophan in tomatoes using multi-walled carbon nanotubes and poly (sulfosalicylic acid) film modified graphite rod electrode. Biosens. Bioelectron. X.

[197] Liu D., Li M., Li H., Li C., Wang G., Li P., Yang B. (2021). Core-shell Au@Cu_2_O-graphene-polydopamine interdigitated microelectrode array sensor for in situ determination of salicylic acid in cucumber leaves. Sens. Actuators B Chem..

[198] Cai Y., Liang B., Chen S., Zhu Q., Tu T., Wu K., Cao Q., Fang L., Liang X., Ye X. (2020). One-step modification of nano-polyaniline/glucose oxidase on double-side printed flexible electrode for continuous glucose monitoring: Characterization, cytotoxicity evaluation and in vivo experiment. Biosens. Bioelectron..

[199] Wang L., Cheng Y., Lamb D., Megharaj M., Naidu R. (2019). Application of Ion Selective Electrode array to simultaneously determinate multi-free ions in solution. Environ. Technol. Innov..

[200] Atas H.B., Kenar A., Tastekin M. (2020). An electronic tongue for simultaneous determination of Ca^2+^, Mg^2+^, K^+^ and NH_4_^+^ in water samples by multivariate calibration methods. Talanta.

[201] Yaroshenko I., Kirsanov D., Kartsova L., Sidorova A., Borisova I., Legin A. (2015). Determination of urine ionic composition with potentiometric multisensor system. Talanta.

[202] Dimeski G., Badrick T., John A.S. (2010). Ion Selective Electrodes (ISEs) and interferences—A review. Clin. Chim. Acta.

[203] Xu Z., Chen J., Liu Y., Wang X., Shi Q. (2022). Multi-emission fluorescent sensor array based on carbon dots and lanthanide for detection of heavy metal ions under stepwise prediction strategy. Chem. Eng. J..

[204] Huang S.-F., Shih W.-L., Chen Y.-Y., Wu Y.-M., Chen L.-C. (2021). Ion composition profiling and pattern recognition of vegetable sap using a solid-contact ion-selective electrode array. Biosens. Bioelectron. X.

